# Impact of the COVID-19 National Lockdown on a Rural and Tribal Population of Tamil Nadu, Southern India: A Mixed-Methods Survey

**DOI:** 10.4269/ajtmh.21-1139

**Published:** 2022-03-16

**Authors:** Rohan Michael Ramesh, Kumudha Aruldas, Sam David Marconi, Venkateshprabhu Janagaraj, Anuradha Rose, Sushil Mathew John, Mahesh Moorthy, Jayaprakash Muliyil, Puthupalayam Kaliappan Saravanakumar, Sitara Swarna Rao Ajjampur, Kulandaipalayam Natarajan Sindhu

**Affiliations:** ^1^ The Wellcome Trust Research Laboratory, Division of Gastrointestinal Sciences, Christian Medical College, Vellore, Tamil Nadu, India;; ^2^ Department of Community Health, Christian Medical College, Vellore, Tamil Nadu, India;; ^3^ Low Cost Effective Care Unit, Christian Medical College, Vellore, Tamil Nadu, India;; ^4^ Department of Clinical Virology, Christian Medical College, Vellore, Tamil Nadu, India

## Abstract

We assessed the impact of the national lockdown on a rural and tribal population in Tamil Nadu, southern India. A mixed-methods approach with a pilot-tested, semi-structured questionnaire and focus group discussions were used. The impact of the lockdown on health, finances, and livelihood was studied using descriptive statistics. Multivariable logistic regression was carried out to identify factors associated with households that borrowed loans or sold assets during the lockdown, and unemployment during the lockdown. Of the 607 rural and tribal households surveyed, households from comparatively higher socioeconomic quintiles (adjusted odds ratio [aOR], 1.84; 95% CI, 1.01–3.34), with no financial savings (aOR, 2.91; 95% CI, 1.17–7.22), and with larger families (aOR, 1.76; 95% CI, 1.22–2.53), took loans or sold assets during the lockdown. Previously employed individuals from rural households (aOR, 5.07; 95% CI, 3.30–7.78), lower socioeconomic households (aOR, 3.08; 95% CI, 1.74, 5.45), and households with no savings (aOR, 1.78; 95% CI, 1.30–2.44) became predominantly unemployed during the lockdown. Existing government schemes for the elderly, differently abled, and widows were shown to be accessible to 89% of the individuals requiring these schemes in our survey. During the focus group discussions, the limited reach of online classes for schoolchildren was noted and attributed to the lack of smartphones and poor Internet connectivity. Although the sudden, unannounced national lockdown was imposed to flatten the COVID-19 curve, aspects related to livelihood and financial security were affected for both the rural and tribal populations.

## INTRODUCTION

The WHO declared COVID-19 a pandemic on March 11, 2020.^1^ In India, early cases of COVID-19 were reported from February 2020.^2^ Preventive measures in India such as travel restrictions, quarantine enforcement, testing, and contact tracing were exercised. The biggest COVID-19 containment measure implemented by the government of India was the national lockdown on March 22, 2020, followed by lockdown extensions until May 31, 2020.^3^ Mobile phone caller tunes with COVID-19 messages were implemented country-wide to spread awareness on COVID-19.^4^^,^^5^

National lockdowns as a containment measure saw growing unemployment and loss of income across all socioeconomic strata, particularly in developing countries.^6^^–^^9^ A multi-state rural study in India reported that, during the lockdown, households had lower income, consumed less food, and when relief through rations or cash transfers became limited, many turned to loans to meet expenses.^9^ In the state of Tamil Nadu, households with a ration card (a government-issued document for eligible households to purchase subsidized food from the Public Distribution System under the National Food Security Act) were eligible for free monthly rations along with a special cash provision of 1,000 Indian rupees (14 U.S. dollars [USD]) during the lockdown.^10^ However, the impact of the COVID-19 national lockdown and the benefit of these financial schemes, especially in rural and difficult-to-reach tribal populations in India has not been well documented. This study was conducted to document the impact of the national lockdown on health, finances, and livelihoods in rural and tribal households of Tamil Nadu.

## MATERIALS AND METHODS

A mixed-methods approach with a pilot-tested, semi-structured questionnaire and focus group discussions (FGDs) was used in a previously censused population in a rural area and a tribal area in Tamil Nadu.^11^ The survey was conducted between July and August 2020 after the easing of lockdown restrictions in a phased manner from June 2020.^12^ The FGDs were conducted in August 2020, after the survey.

### Settings and participants.

The study was conducted in the Timiri and Jawadhu hills blocks of the Vellore and Tiruvannamalai districts, respectively. Both study populations were censused in 2017.^13^ Timiri, a rural block of 219 villages, has a population of 105,691.^14^ The health needs are catered to by four government primary health centers and 25 health sub-centers.^15^ More than 42% of the adults are employed as daily-wage laborers, 20% as agricultural workers, and 15% as skilled and 13% as semi-skilled laborers. Eighteen percent of adults are uneducated and 9% belong to the lowest wealth index quintile. Jawadhu hills is a reserve forest located ∼760 m above sea level and has around 390 villages overlapping the Vellore and Tiruvannamalai districts in the northern part of Tamil Nadu.^16^ This census covers a population of 51,999 people residing in 154 villages comprised of smaller hamlets. Three primary health centers and 13 health sub-centers provide health care to this population.^15^ A large section of the population is agrarian (88%) with a small percentage (2.7%) migrating seasonally to the nearby districts for semi-skilled work. About half the adults have not received any formal education and 58% belong to the lowest wealth index quintile.

### Questionnaire-based community survey.

A sample size of 609 households (both rural and tribal) was estimated using the proportion of families that received loans during the lockdown as estimated in a recent study in rural India (33%; relative precision, 20%; α, 0.05; design effect, 2.5; assuming a non-response rate of 20% in this study population).^9^ A stratified multistage sampling technique was used to select households in each of the blocks. Simple random sampling was carried out to select six clusters from the Timiri block and four clusters from the Jawadhu block. In each selected cluster, three villages were selected using probability proportional to the size, and approximately 20 individuals from each of the selected villages were chosen randomly from the census list to achieve the required sample size.

The head of the household or an adult member from each household was invited to take the survey. The questionnaire was administered by an interviewer in the local language using an android-based mobile platform (SurveyCTO; Dobility, Inc., Cambridge, MA, and Ahmedabad, India).^17^ The survey was conducted in adherence to strict COVID-19 precautions.

### Focus group discussions.

FGDs were conducted to understand how the rural and tribal communities were affected by the lockdown in various domains, including access to health care, food security/relief, schooling, financial challenges, and impact on livelihood. Four FGDs, two among men and two among women, were conducted in the Timiri block and Jawadhu hills. For each FGD, one key informant from six randomly selected villages were selected purposefully and invited to represent their respective villages in the FGD. Not more than six key informants were included in each FGD to maintain physical distancing while being seated in a wide circle. Thus, 24 participants, 12 men and 12 women, were included in the FGDs. An interviewer and scribe conducted the FGDs in Tamil (the local language spoken in the state of Tamil Nadu). All informants were given an opportunity to talk about their villages. The lead facilitator, while acknowledging everyone’s contribution to the discussion, posed broad questions in all domains of impact to each participant. All the FGDs were audio-recorded, transcribed verbatim, and translated into English.

### Statistical analysis.

Baseline sociodemographic details of the selected household head/individuals were extracted from a census conducted in 2017. The exposure variables studied included age, gender, area of residence, education, marital status, socioeconomic quintiles, possession of a mobile phone/television, family size, and savings. The wealth index was assessed using quintiles (1 being low and 5 being high), derived from household assets using principal component analysis as described elsewhere.^18^ The outcome variables assessed included the procurement of loans and/or assets sold, and the employment status of individuals in the household during the lockdown, using independent multivariate models. Univariable logistic regression for each of the outcome variables was performed, and significant variables (*P* < 0.05) were included in the multivariable logistic regression using backward elimination. Analysis was done using STATA version 16.0 (StataCorp LLC, College Station, TX) and RStudio (Desktop 1.4.1103; RStudio, Boston, MA) For FGDs, using an a priori thematic code list, the transcripts were coded and analyzed using ATLAS.ti 8.4.4 software.

### Ethical considerations.

The study was approved by the institutional review board of Christian Medical College, Vellore (no. 12976). Written informed consent was obtained from the head or adult member of the household participating in the survey. A separate written informed consent was obtained from FGD participants (key informants) for participation in and audio-recording of the discussion.

## RESULTS

Of the 611 households approached, 607 consented to participate in the survey, with 401 (66.1%) and 206 (33.9%) households from Timiri and Jawadhu hills, respectively. The characteristics of the Timiri and Jawadhu households are shown in Table 1. The majority of the respondents were 30 to 50 years old. About half (54.9%) of the Jawadhu hills respondents were uneducated and belonged to households with the lowest wealth index quintile (57.8%) (Table 1).

**Table 1:** Characteristics of the survey respondents in rural (Timiri) and tribal (Jawadhu hills) households (*N* = 607)

Variable	Rural, *n *(%); (*n* = 401)	Tribal, *n *(%); (*n* = 206)	Overall, *n* (%)
Age, y			
18–30	55 (13.7)	45 (21.9)	100 (16.5)
31–40	99 (24.7)	66 (32)	165 (27.2)
41–50	98 (24.4)	44 (21.4)	142 (23.4)
51–60	68 (17)	33 (16)	101 (16.6)
> 60	81 (20.2)	18 (8.7)	99 (16.3)
Gender			
Male	190 (47.4)	119 (57.8)	309 (50.9)
Female	211 (52.6)	87 (42.2)	298 (49.1)
Education			
College, diploma, university	21 (5.2)	9 (4.4)	30 (5)
Higher secondary school	26 (6.5)	5 (2.4)	31 (5.1)
High school	74 (18.5)	13 (6.3)	87 (14.3)
Middle school	94 (23.4)	23 (11.2)	117 (19.3)
Primary school	132 (32.9)	43 (20.9)	175 (28.8)
Uneducated	54 (13.5)	113 (54.9)	167 (27.5)
Socioeconomic quintile*			
5	107 (26.7)	1 (0.5)	108 (18)
4	101 (25.2)	3 (1.5)	104 (17.1)
3	105 (26.2)	11 (5.3)	116 (19.1)
2	51 (12.7)	72 (34.9)	123 (20.2)
1	37 (9.2)	119 (57.8)	156 (25.7)
Family size			
≤ 4	257 (64.1)	117 (56.8)	374 (61.6)
≥ 5	144 (35.9)	89 (43.2)	233 (38.4)
Type of phone, if owned by the household			
Basic phone	177 (44.1)	87 (42.2)	264 (43.5)
Smartphone	73 (18.2)	22 (10.7)	95 (15.7)
No phone	151 (37.7)	97 (47.1)	248 (40.9)

* Quintiles: 1 = low and 5 = high.

### Impact of the national lockdown.

The findings from the survey are highlighted, along with the key findings from the FGDs, with reference to each domain studied.

#### Access to health care.

Family members in ∼25% (150 of 607) of the households required monthly medications for chronic medical conditions such as hypertension (88 of 150, 59%) and/or diabetes (77 of 150, 51%). During the lockdown, 42% of them were able to procure the medications from government health centers (data not tabulated). However, 11% of these patients had to go outside the village to procure the drugs, with 20% of them asking somebody else to procure their medication for them. About 7% of households (43 of 607) reported that at least one family member required emergency medical assistance during the lockdown, and 47% (20 of 43) expressed that the lockdown did not affect their access to medical services. Difficulties experienced included lack of transport (44%), inability to leave the house because of the lockdown (12%), and closure of health centers (7%). Of the 19 pregnant women in the 607 households, 17 (90%) reported that their antenatal checkups remained unaffected, with only two mothers facing transportation issues.

The FGDs for both Timiri and Jawadhu hills corroborated these findings from the survey (Table 2). Recurring themes from the FGDs were lack of public transportation; inflated private transport fares; suspension of government-run mobile clinics, which included health services for chronic illnesses for the elderly; and unavailability of doctors in health centers (doctors were deployed elsewhere for COVID emergency duty).

**Table 2:** Highlights from the focus group discussions to study the impact of the national lockdown among rural (Timiri) and tribal (Jawadhu hills) households

Domain	Highlights that emerged from the focus group discussions
Maternal and child health services	Men (Timiri): “The nurses are continuing to come to our village to vaccinate newborns. They directly go to the homes and give the vaccine.”
	Men (Jawadhu hills): “If pregnant women have to deliver at a hospital, they have to go to Jamunamaruthur.* That is not a problem, as ambulance will come in when called [108 ambulance services].”
	Women (Jawadhu hills): “There is no problem in going for delivery. If we call an ambulance, it will come. But we don’t get the cell phone tower signal to call them. So, it will be difficult to call the ambulance. If we climb to the top of the hill, we get signal, but it will become late.”
General health services	Men (Timiri): “Mobile van was coming every month, before corona, to give tablets and injections for the diabetics every month. Due to corona, this has stopped.”
	Women (Timiri): “If we ask why the doctor didn’t come, they [health center staff] tell us that doctors are away on corona duty.”
	Women (Jawadhu hills): “In our village a nurse would come and check BP [blood pressure] . . . they will give tablets. Nurse is not coming for 2 to 3 months. Someone has to take their notebook [of treatment history of chronic illness] to the hospital and get the tablet. They are not going for checkup because they cannot walk.”
Anganwadi center services, midday meal scheme	Women (Timiri): “A child who otherwise did not eat well would eat well by themselves when they sit with other children to eat at the *anganwadi*. It is now very difficult to manage them at home and make them eat. They teach ‘one, two, three, and ABCD’ at *anganwadi*. Now there is no learning as well.”
	Men (Jawadhu hills): “They gave food for children aged more than 3 years and health mix for those below 3 years. Now, because of corona, they (*anganwadi* teacher and helper) come only once in 15 days to give the health mix to every house.
Schooling	Women (Timiri): “Now children are attending schools by taking online classes. Children do not concentrate as they have to watch the screen continuously for [1 to 1.5] hours. They start watching something else other than the teaching. All these things are affecting children’s minds . . . . Children cannot maintain physical distance between themselves. They will stay together and talk. They cannot wear masks for a long time. So, it is good for children to be at home in the current situation . . . .
	Men (Jawadhu hills): “Schooling is now online. They don’t get Internet network on the hills. Hence, we will have to take them to Jamunamaruthur and leave the children in a relative’s house to study. We are unable to do this every day.”
	Women (Jawadhu hills): “Following closure of schools, girl children are made to do household chores and are unable to study. Because of corona, their education is being affected.”
Government ration supplies	Women (Timiri): “Before corona, they were giving one oil packet and 1 kg dhal for 30 rupees. For the last 4 months, they are giving sugar, oil, and everything for free . . . . Rice is always free . . . . We were getting extra 5 kg rice per person for free.”
	Men (Jawadhu hills): “The government gave 1,000 rupees per ration card at ration shops because we had no income.”
Agriculture	Men (Timiri): “An agricultural cooperative bank was started by the government for farmers. Farmers got 20,000 to 100,000 rupees as loans from these banks. The loan re-payments are now due. We previously could extend the loan period without any interest, but now we are being asked to pay when we are under loss. So, why farmers will not die [referring to previous farmers’ suicide for their inability to pay loans]?”
	Men (Jawadhu hills): “Cultivation of crops was not affected due to lockdown. However, fertilizer costs have gone up. We have cultivated vegetables, but there is no transport during this lockdown to take it to the market downhill. The market is also closed, so no one to buy them.”
Business/employment	Men (Timiri): “We are not able to go anywhere for work. We are very much affected.”
	Women (Timiri): “We have taken loans from many places . . . . Due to corona and the lockdown, we are not able to earn and repay the loan. Now, they are asking us to re-pay the loan with interest for these 4 months. We don’t even have a way for food. How will we pay the interest? The money lender is coming and asking us for money every day.”
	Men (Jawadhu hills): “If you see my village people, 75% of them were going for work outside to cities like Chennai and Bengaluru. Only 25% do farming. After lockdown, because of the corona, all are facing difficulties as they are not able to go out for work.”
	Women (Jawadhu hills): “Not everyone cultivates. Only those who have land cultivate. What will the other people who don’t have land do?”
“Was the lockdown necessary?”	Men (Timiri): “Definitely. We need the lockdown for the safety of the people.”
	Women (Jawadhu hills): “It is needed because if there was no lockdown, people will go from here to another place and will get infected by corona. So, this lockdown is definitely needed.”

* Primary health centers offering maternal and child health services are located at Jamunamaruthur that may or may not be connected to the surrounding villages by roads.

#### Food security and relief.

The survey found that 92% of the households (560 of 607) had ration cards. Almost all households with ration cards (99%) received food rations and a one-time payment of 1,000 Indian rupees (∼14 USD) as a lockdown relief measure.^19^ However, 40% of the households felt that the ration quantity provided was insufficient, given that 47% of the households were completely dependent on government ration supplies and had no income because of unemployment. Some households (13%) also received supplies from nongovernmental organizations operating in the area. Of the 150 households (24.7%) availing themselves of specific government schemes (old age, disability, widow pensions) before the lockdown, 89% (134 of 150) reported no disruption in these services during the lockdown.

These findings were reiterated during the FGDs. Participants stated that ration shops continued to provide supplies such as rice, pulses, sugar, salt, and kerosene (domestic stove fuel) to eligible families, free of cost, during the lockdown (previously provided at subsidized rates), along with the special cash relief fund (Table 2). It was strongly felt that these measures were extremely helpful to financially deprived households during the lockdown. However, these incentives were provided only for first 3 to 4 months of the lockdown. *Anganwadis* (informal government-run education centers for preschool children under the Integrated Child Development Scheme) supplying nutritional packages to beneficiaries weekly/fortnightly despite the lockdown was much appreciated. The midday meals scheme was modified, and schools supplied each child’s food quota (grains and eggs), which was collected by parents on a weekly basis and shared among other family members during the lockdown.

#### Schooling.

Schools and *anganwadis* in Tamil Nadu were closed on March 16, 2020.^20^ Although the survey did not address aspects related to schooling, the FGDs revealed several issues. FGD participants appreciated the informal education that was usually provided to preschoolers by *anganwadis*, and expressed concern that preschoolers were now missing out on this because of the lockdown (Table 2). It was felt that school closures during the lockdown affected children’s academics, although online teaching was initiated after government protocol. Although children in the 11th grade or higher were provide laptop computers by the government, children in lower grades were left with no option but to use their parents’ smartphones to connect to online classes. Many families found smartphones and laptops unaffordable. As corroborated in the survey, only 14.7% of the households owned a smartphone, whereas 43.5% owned only a basic phone and 40.9% did not own any phone at all (Table 1). When the two blocks were compared, 18.2% of the households owned a smartphone in the rural Timiri block whereas only 10.7% of households in the tribal block of Jawadhu hills owned a smartphone. Children in the neighborhood attended online classes together using a single phone, ignoring physical distancing norms. Furthermore, poor network connectivity in Jawadhu hills affected attendance to these online classes. Overall, online teaching was described as not sufficiently engaging, with poor concentration showed by children. Furthermore, female children were kept engaged with household chores during online classes, because they were then at home. Those who did have access to the Internet via smartphones or laptops to attend online classes felt that the increased screen time and access to non-educational, unsupervised content on the Internet was detrimental to their child’s learning. Furthermore, the absence of the school routine brought disruption in terms of sleep and food discipline, with an increase in unintentional childhood injuries being reported.

#### Loans acquired and/or assets sold during the lockdown.

About 32% of the households (195 of 607) took out loans during the lockdown, more so in Timiri (36%) compared with Jawadhu hills (24%) (data not presented). Money was borrowed from local money lenders (33%), pawnbrokers (32%), family and friends (24%), self-help groups (16%), and banks (12%). Twenty-six percent of the households had financial savings, such as bank savings (39%), chit funds (38%), home savings (29%), or post office schemes (9%). A small number of households (88 of 607) sold assets such as livestock (12%) and gold jewelry (3%) during the lockdown. Most households (187 of 195) that took out loans during the lockdown were finding it difficult to repay the loans. Procuring vegetables/provisions (66%) and potable water (7%), and paying electricity bills (18%) and school fees (3%) became challenging (data not presented). Households from the upper socioeconomic quintiles (adjusted odds ratio [aOR], 1.84; 95% CI, 1.01–3.34), with no financial savings (aOR, 2.91; 95% CI, 1.17–7.22), large families (more than five members) (aOR, 1.76; 95% CI, 1.22–2.53); and those that perceived themselves to be severely affected by the lockdown (aOR, 4.64; 95% CI, 2.29–9.40) were more likely to seek loans or sell assets during this period (Table 3).

**Table 3:** Factors associated with the procurement of loans and/or assets being sold among rural (Timiri) and tribal (Jawadhu hills) households during the national lockdown (*N* = 607)

Variable	*n* (%)	Loan taken or assets sold		Unadjusted OR (95% CI)	Adjusted OR (95% CI)
		Yes, *n *(%); (*n* = 249, 41%)	No, *n *(%); (*n* = 358, 59%)		
Age of head of household, y					
> 60	149 (24.5)	48 (32.2)	101 (67.8)	Ref.	–
51–60	132 (21.7)	49 (37.1)	83 (62.9)	1.24 (0.76–2.03)	–
41–50	156 (25.7)	74 (47.4)	82 (52.6)	1.90 (1.19–3.02)	–
31–40	131 (21.6)	62 (47.3)	69 (52.7)	1.89 (1.16–3.07)	–
23–30	39 (6.4)	16 (41)	23 (59)	1.46 (0.71–3.02)	–
Gender, head of household					
Female	100 (16.5)	30 (30)	70 (70)	Ref.	–
Male	507 (83.5)	219 (43.2)	288 (56.8)	1.77 (1.12–2.82)	–
Area					
Tribal (Jawadhu)	206 (33.9)	77 (37.4)	129 (62.6)	Ref.	–
Rural (Timiri)	401 (66.1)	172 (42.9)	229 (57.1)	1.26 (0.89–1.78)	–
Education, head of household					
College, diploma, university	21 (3.4)	11 (52.4)	10 (47.6)	Ref.	Ref.
Higher secondary school	21 (3.4)	6 (28.6)	15 (71.4)	0.36 (0.10–1.30)	0.35 (0.91–1.35)
High school	113 (18.6)	62 (54.9)	51 (45.1)	1.10 (0.43–2.81)	1.16 (0.43–3.08)
Middle school	107 (17.6)	46 (43)	61 (57)	0.68 (0.27–1.75)	0.72 (0.27–1.93)
Primary school	146 (24.1)	60 (41.1)	86 (58.9)	0.63 (0.25–1.59)	0.54 (0.20–1.43)
Uneducated	199 (32.8)	64 (32.2)	135 (67.8)	0.43 (0.17–1.07)	0.42 (0.16–1.15)
Socioeconomic quintile*					
5	108 (17.8)	43 (39.8)	65 (60.2)	Ref.	Ref.
4	104 (17.1)	58 (55.8)	46 (44.2)	1.91 (1.10–3.29)	1.84 (1.01–3.34)†
3	116 (19.1)	48 (41.4)	68 (58.6)	1.07 (0.63–1.82)	0.99 (0.55–1.78)
2	123 (20.3)	50 (40.7)	73 (59.3)	1.03 (0.61–1.75)	1.05 (0.57–1.93)
1	156 (25.7)	50 (32)	106 (68)	0.71 (0.43–1.19)	0.80 (0.44–1.48)
Savings					
Personal savings and government schemes	28 (4.6)	8 (28.6)	20 (71.4)	Ref.	Ref.
Personal savings only	129 (21.3)	35 (27.1)	94 (72.9)	0.93 (0.38–2.31)	0.98 (0.37–2.28)
Government schemes only	122 (20.1)	42 (34.4)	80 (65.6)	1.31 (0.53–3.23)	1.82 (0.69–4.80)
None of the above	328 (54)	164 (50)	164 (50)	2.50 (1.07–5.84)	2.91 (1.17–7.22)†
Family size					
≤ 4	374 (61.6)	133 (35.6)	241 (64.4)	Ref.	Ref.
≥ 5	233 (38.4)	116 (49.8)	117 (50.2)	1.80 (1.29–2.51)	1.76 (1.22–2.53)†
Perceived impact of lockdown by the household					
Not or mildly affected	60 (9.9)	13 (21.7)	47 (78.3)	Ref.	Ref.
Moderately affected	276 (45.5)	94 (34.1)	182 (65.9)	1.87 (0.96–3.62)	2.26 (1.11–4.59)†
Severely affected	271 (44.7)	142 (52.4)	129 (47.6)	3.98 (2.06–7.69)	4.64 (2.29–9.40)‡

OR = odds ratio; Ref. = reference value. The survey respondent from each household was considered to represent the respective household.

* Quintiles: 1 = low and 5 = high.

† Significant at *P* < 0.05.

‡ Significant at *P* < 0.001.

The FGDs corroborated these findings; many households took out loans to meet expenses during the lockdown (Table 2). Families that had secured loans previously were in debt given their lack of income, placing them at potential risk of harassment from lenders (a common problem encountered routinely with informal loaning in these communities) (Table 2).

#### Unemployment.

**Figure 1.**
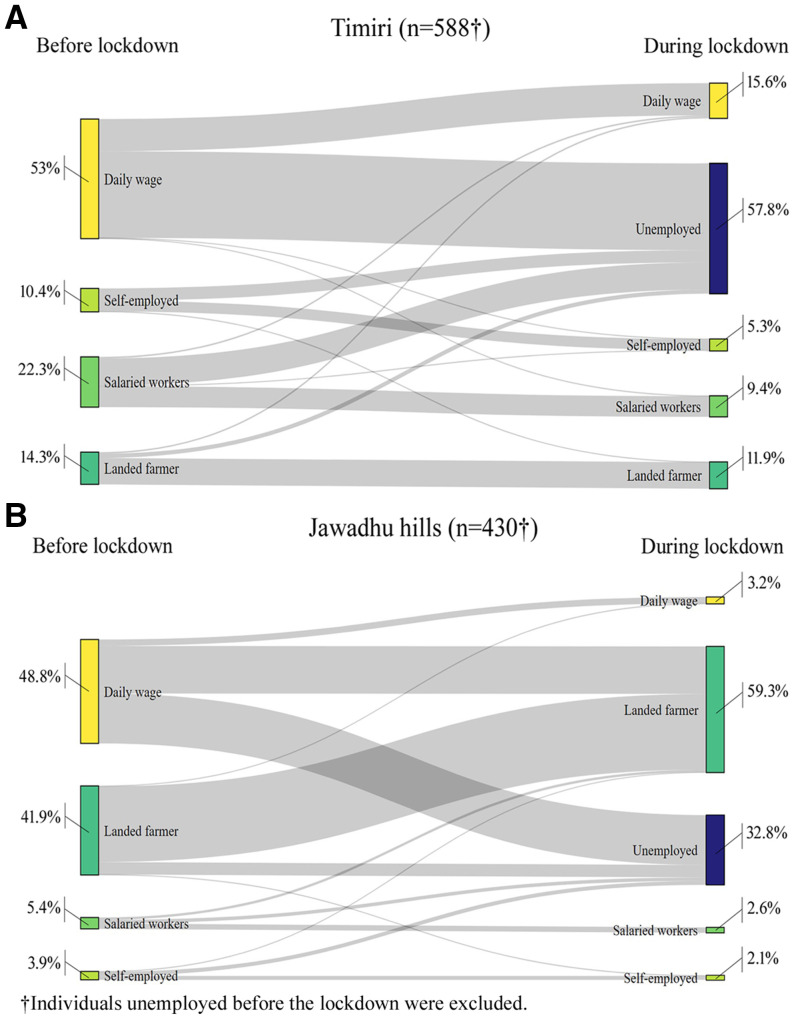
Sankey diagram depicting the transition between various occupational groups among previously employed individuals before and during the lockdown in (**A**) rural (Timiri) and (**B**) tribal (Jawadhu hills) households (*N* = 1,018). This figure appears in color at www.ajtmh.org.

**Figure 2.**
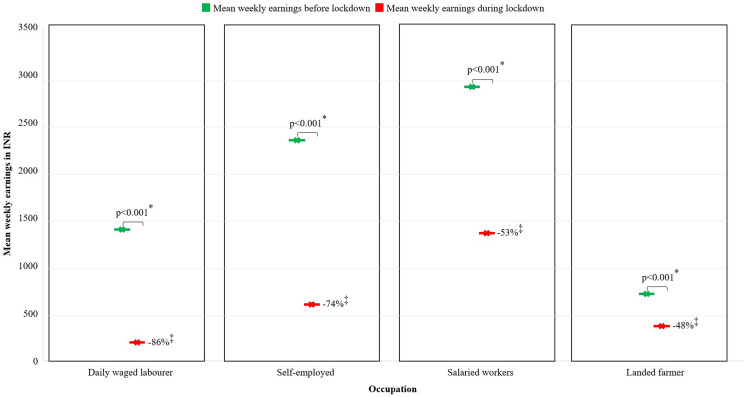
Impact of the national lockdown on the weekly earnings (in Indian rupees [INR]) of previously employed individuals in rural (Timiri) and tribal (Jawadhu hills) households among various occupations (*N* = 1018†). * Paired *t*-test. † Individuals unemployed before the lockdown were excluded. ‡ Percentage decrease in weekly earnings. This figure appears in color at www.ajtmh.org.

About 47% of those previously employed (481 of the 1,018 working individuals from the 607 households surveyed) became unemployed during the lockdown. Unemployment was higher in Timiri (58%) compared with Jawadhu hills (33%) (Figure 1). In addition, a 66% decline in weekly wages was noted among individuals who continued to stay employed. Although there was a significant decrease in mean weekly wages among all occupational groups after lockdown, daily-wage workers were the most affected (86% decline in weekly wages) (Figure 2). During the lockdown, most daily-wage workers in Timiri became unemployed (72.4%) compared with Jawadhu hills (48.1%). In Jawadhu hills, 45.7% of daily-wage workers resorted to farming during the lockdown (Figure 1). Previously employed individuals from households with lower wealth index quintiles had significantly greater odds of being unemployed during the lockdown (OR, 3.08; 95% CI, 1.74–5.45) (Table 4). Individuals with no savings (aOR,1.78; 95% CI, 1.30–2.44) and those from Timiri (aOR: 5.07, 95% CI, 3.30–7.78) were also more likely to be unemployed during the lockdown.

**Table 4:** Factors associated with unemployment during the national lockdown among previously employed individuals in rural (Timiri) and tribal (Jawadhu hills) households (*N* = 1,018)

Variable	*n* (%)	Unemployment during lockdown		Unadjusted OR (95% CI)	Adjusted OR (95% CI)
		Yes, *n* (%); (*n* = 481, 47%)	No, *n* (%); (*n* = 537, 53%)		
Age, y					
14–30	259 (25.4)	133 (51.4)	126 (48.6)	Ref.	–
31–40	282 (27.7)	125 (44.3)	157 (55.7)	0.75 (0.54–1.06)	–
41–50	221 (21.7)	95 (43)	126 (57)	0.71 (0.50–1.02)	–
51–60	155 (15.2)	70 (45.2)	85 (54.8)	0.78 (0.52–1.16)	–
> 60	101 (9.9)	58 (57.4)	43 (42.6)	1.28 (0.80–2.03)	–
Gender					
Male	608 (59.7)	285 (46.9)	323 (53.1)	Ref.	–
Female	410 (40.3)	196 (47.8)	214 (52.2)	1.04 (0.81–1.33)	–
Area					
Tribal (Jawadhu)	430 (42.2)	141 (32.8)	289 (67.2)	Ref.	Ref.
Rural (Timiri)	588 (57.8)	340 (57.8)	248 (42.2)	2.81 (2.18–3.64)	5.07 (3.30–7.78)†
Education					
College, diploma, university	79 (7.8)	38 (48.1)	41 (51.9)	Ref.	–
Higher secondary school	64 (6.3)	32 (50)	32 (50)	1.08 (0.56–2.09)	–
High school	192 (18.9)	95 (49.5)	97 (50.5)	1.06 (0.63–1.78)	–
Middle school	164 (16.1)	88 (53.6)	76 (46.4)	1.24 (0.73–2.14)	–
Primary school	180 (17.7)	89 (49.4)	91 (50.6)	1.05 (0.62–1.79)	–
Uneducated	339 (33.3)	139 (41)	200 (59)	0.75 (0.46–1.23)	–
Marital status					
Married	808 (79.4)	374 (46.3)	434 (53.7)	Ref.	–
Unmarried	125 (12.3)	65 (52)	60 (48)	1.26 (0.86–1.83)	–
Separated, divorced, widowed	85 (8.4)	42 (49.4)	43 (50.6)	1.13 (0.72–1.77)	–
Socioeconomic quintile*					
5	143 (14)	72 (50.3)	71 (49.7)	Ref.	Ref.
4	183 (18)	106 (57.9)	77 (42.1)	1.36 (0.87–2.11)	1.34 (0.86–2.09)
3	191 (18.8)	97 (50.8)	94 (49.2)	1.02 (0.66–1.57)	1.10 (0.70–1.72)
2	236 (23.2)	85 (36)	151 (64)	0.55 (0.36–0.85)	1.41 (0.84–2.38)
1	265 (26)	121 (45.7)	144 (54.3)	0.83 (0.55–1.24)	3.08 (1.74–5.45)†
Savings for daily living					
Yes	241 (23.7)	90 (37.3)	151 (62.7)	Ref.	Ref.
No	777 (76.3)	391 (50.3)	386 (49.7)	1.70 (1.26–2.29)	1.78 (1.30–2.44)†
Family size					
≤ 4	533 (52.4)	248 (46.5)	285 (53.5)	Ref.	–
≥ 5	485 (47.6)	233 (48)	252 (52)	1.06 (0.83–1.36)	–

OR = odds ratio; Ref. = reference value. All employed members of the household were included. Those unemployed before the lockdown were excluded.

* Quintiles: 1 = low and 5 = high.

† Significant at *P* < 0.001.

FGDs indicated that livelihood for most households was affected during the lockdown. Farmers were unable to sell their produce in markets because markets were closed. An inability to procure loans to purchase farming supplies (seeds, fertilizers, pesticides), inflated rates of farming supplies, and non-deferment of previous loans aggravated the crisis (Table 2). People involved in small-scale dairy farms, flour mills, theaters, and travel agencies; and barbers, weavers, mechanics, brick-kiln workers, and autorickshaw drivers were affected. Commuting to cities and neighboring states for work was challenging because of the lack of transport during the lockdown, which worsened the financial status in many households. However, the survey revealed that 91.8% of the respondents (98.1% in Jawadhu hills and 88.5% in Timiri) felt that the lockdown imposed by the government was indeed necessary to curtail the increase in COVID-19 cases.

## DISCUSSION

The national lockdown affected various aspects of life among the rural and tribal households, as indicated by this mixed-methods survey. We highlight issues that warrant attention during the ongoing pandemic.

Essential health-care services in both the rural and tribal areas surveyed were minimally disrupted. It is to be acknowledged and appreciated that routine maternal and child health services through sub-centers and *anganwadis* continued in both the surveyed rural and tribal areas. Similar findings have been documented in a cross-sectional study conducted around the same period in India.^21^ The lack of transport, strict travel restrictions, and inflated transport fares were notable challenges faced by the elderly with chronic illnesses. In another Indian survey, only one quarter of those with a chronic disease was able to access health services.^22^ A planned, strategized distribution of chronic illness medications using community nurses, health aides, and other community volunteers, in coordination with primary health centers/sub-centers would be a more pragmatic approach during lockdowns. States such as Kerala, Odisha, and Rajasthan were able to engage such cadres to work in the community and take up pandemic-related relief activities.^23^ Provision of limited, yet planned, public transport for patients needing regular health-care access and the assurance that at least one doctor is available at remote locations are suggested.

Households with ration cards were supported with a free supply of groceries for 3 to 4 months during the lockdown, and that has been the case in the other states too.^9^^,^^24^ Although the government’s cash-based financial assistance provided to households having a ration card with no income during the lockdown was a notable relief, it seemed insufficient because employment was low as a result of the strictly enforced norms, with no additional financial savings by the majority to cope with the lockdown. Financial assistance provided to the disadvantaged (elderly, widowed, and differently abled) via government schemes that were uninterrupted during the lockdown is commendable. Efforts by *anganwadis* and schools to distribute nutritional packages to beneficiaries during the lockdown were also noteworthy. However, the real impact of these measures on the benefactor remains questionable because supplies, especially food, were often shared with other household members.

Respondents in Timiri and Jawadhu felt education for schoolchildren was considerably affected during the lockdown. In the absence of physical schooling, Internet-based facilitated learning via online virtual platforms was implemented by the schools that had the facilities to conduct these classes. Only 14.5% of our surveyed households owned a smartphone, which is the minimum prerequisite to access an online educational platform, thus limiting the reach of online learning in these communities. A national survey also reported common issues deterring online education such as unavailability of a smartphone, network/connectivity issues, erratic electricity, child not able to learn remotely, and child not interested or too young to operate a device without parental guidance.^25^ In our setting, poor network connectivity in remote areas, sharing of the device by many children, household chores that became a burden for female children, and concern regarding unsupervised access to non-educational content were additional challenges reported by parents. With no other alternative available, these communities had to rely on printed educational material provided by the schools, which only 55.4% of children from grades 1 to 8 in Tamil Nadu received when schools were closed, as reported by the national survey. Classes broadcast through government television networks (the majority of the homes in the state of Tamil Nadu have a government-provided television) may be a pragmatic alternative to online classes. A good example of this comes from the neighboring state of Kerala, where 77% of the households had access to a school educational channel broadcasted on a government cable TV network during the lockdown.^26^

Nearly, one third of the households took out loans or sold assets during the lockdown. Our study highlighted the fact that families with a comparatively higher socioeconomic status were more likely to take out loans from local lenders/agencies. This was perhaps because of greater confidence in the ability to secure employment after the easing of the lockdown. Larger families took loans or sold assets, with expenses proportional to family size. Furthermore, those who took out loans or sold assets perceived the lockdown to have a moderate to a negative impact on their lives, compelling them to borrow money or sell assets as a result of their looming financial insecurity.

About half of the previously employed individuals became unemployed during the lockdown. This is similar to findings from a multi-state study in rural India where 56% lost employment during the lockdown.^9^ Although daily-wage laborers in the tribal block of Jawadhu hills could take up farming as an alternative, a large proportion became unemployed, with no other option on which to rely. A mean decline of 66% in weekly individual earnings during the lockdown noted in our survey is similar to the 63% decline documented by a previous study in rural India.^9^ Robust financial relief measures focused on lower socioeconomic groups would immensely benefit rural households in particular.

The proportion of polymerase chain reaction-confirmed cases of COVID-19 until July 30, 2020 (when the survey happened) in the districts where the surveyed blocks were located was 0.15% (5,875 of 39,36,331) and 0.25% (6,052 of 24,64,875) in the Vellore and Tiruvannamalai districts, respectively, and increased to 0.45% (17,899 of 39,36,331) and 0.71% (17,619 of 24,64,875), respectively in the three subsequent months.^27^^–^^29^ Although the public health relevance of implementing a lockdown has been documented, the long-term impact on access to routine health care and education, and the impact on household finances and livelihoods need to be weighed carefully.^30^ Most respondents in our study agreed that the lockdown was a necessary measure to curb the increase in COVID-19 cases, despite the significant impact on health, education, and employment.

This survey is strengthened by our approach of using a mixed-methods (quantitative and FGD) survey conducted in person, as opposed to other surveys on COVID-19 pandemic impact using phone/online surveys, which lead to the exclusion of rural and tribal households without phones or with limited access to surveys via online platforms. The survey was limited with reference to the loss-of-income estimation because the information was provided by the respondent on behalf of other household members. Also, because there was no follow-up carried out, the subsequent impact of COVID-19 and additional lockdowns that may have further disadvantaged these households, and additional governmental measures taken to ameliorate this situation, were not estimated.

## CONCLUSION

Although the national lockdown was implemented as an effective measure to curtail the spread of COVID-19, there was a notable impact on routine health-care access, finances, education, and livelihood in the rural and tribal households. The public health benefits versus socioeconomic risks of a sudden lockdown such as the risk of unemployment, loss of household assets, and risk of informal loans with the inability to repay loans have to be weighed carefully. Tailored interventions for vulnerable groups (e.g., large families, those with a low wealth index, families with no savings, daily-wage workers) in these rural and tribal populations need to be planned ahead. This first-ever lockdown in India has shown that we have much to learn, as documented by our study.
